# Development and validation of a low-cost spectrophotometric method for cannabidiol (CBD) quantification based on the Beam reaction

**DOI:** 10.1186/s42238-026-00473-1

**Published:** 2026-07-16

**Authors:** Fresia Melina Silva Sofras, Martín Federico Desimone, Sofia Municoy

**Affiliations:** 1 Universidad de Buenos Aires, Facultad de Farmacia y Bioquímica, Departamento de Ciencias Químicas, Cátedra de Química Analítica Instrumental, Buenos Aires, 1113 Argentina; 2 Centro de Investigación y Extensión Forestal Andino Patagónico, Esquel, 9200 Argentina; 3 CONICET-Universidad de Buenos Aires, Instituto de Química y Metabolismo del Fármaco (IQUIMEFA), Buenos Aires, 1113 Argentina

**Keywords:** Beam reaction, CBD quantification, Low-cost method, UV-Vis spectrophotometry, Validation

## Abstract

Cannabidiol (CBD) is a compound of high pharmacological interest, requiring accessible analytical methods for its identification and measurement. This study proposes the transformation of the classic Beam reaction, traditionally a qualitative colorimetric test, into a robust, low-cost quantitative technique using UV-Vis spectrophotometry. The method is based on the formation of a specific chromophore between CBD/CBDA and a 5% ethanolic KOH solution. Spectral characterization identified a maximum absorbance peak at 530 nm. Kinetic analysis established that the reaction stabilizes after a 24-hour incubation period, ensuring high reproducibility for batch analysis. The optimized protocol demonstrated high linearity (R^2^ > 0.99) and excellent precision, with a Relative Standard Deviation (RSD%) of 2.88% for intraday and 4.74% for interday assays. The limit of detection (LOD) and limit of quantification (LOQ) were 1.84 and 5.58 µg/mL respectively. By utilizing widely available reagents and standard instrumentation, this method provides a significant advantage for laboratories and research centers where high-performance liquid chromatography (HPLC) is not feasible. The findings validate the Beam reaction as a precise, simple, and economically viable tool for the quantification of total CBD.

## Highlights

Quantitative UV–Vis method developed from the classic Beam reaction.

Low-cost, robust approach for CBD/CBDA determination.

Excellent linearity (R² > 0.99) and precision (RSD < 5%).

Practical alternative to HPLC using widely available reagents.

## Introduction

Cannabidiol (CBD) is one of the most prevalent phytocannabinoids found in *Cannabis sativa* L^1–4^. Since its discovery, CBD has been postulated in scientific literature as a molecule of high medicinal value, owing to its broad therapeutic window and lack of psychoactivity-related adverse effects. Accordingly, in 2018, the FDA approved the first cannabis-derived drug, Epidiolex (100 mg/mL CBD) (Patel et al. 2017, Devinsky et al. 2018, Thomas and Cunningham 2018, Hazekamp 2018).

Argentina enacted Law 27,350 in 2017 to authorize the research and medicinal use of cannabis, initially via the importation of a single product: Charlotte’s Web Oil, a hemp derivative (Chemotype III) with high CBD content. While medicinal use was already prevalent among the population, this legal framework catalyzed a massive expansion of an unregulated market of oils claiming to contain “CBD” ^9–11^.

As the demand for CBD-based products, ranging from oils to isolates, continues to grow globally, there is an increasing need for reliable analytical methods to ensure precise dosing and product standardization (Hazekamp 2018, Johnson et al. 2022, Lachenmeier et al. 2024, Miller et al. 2022, Evans 2020, Johnson et al. 2022, Wheeler 2020, Bhamra et al. 2021). Currently, high-performance liquid chromatography (HPLC) and gas chromatography (GC) coupled with mass spectrometry are the gold standards for cannabinoid quantification due to their high sensitivity and specificity (Leghissa et al. 2018, Pourseyed Lazarjani et al. 2020, Pellati et al. 2018, Brighenti et al. 2021, Citti et al. 2018, Carvalho et al. 2023, Sofrás et al. 2023, Sofrás 2023). However, these techniques require expensive equipment, high-purity solvents, and specialized personnel, which limits their accessibility in small-scale laboratories, agricultural settings, or regions with limited infrastructure (Sofrás et al. 2023).

**Fig. 1:**
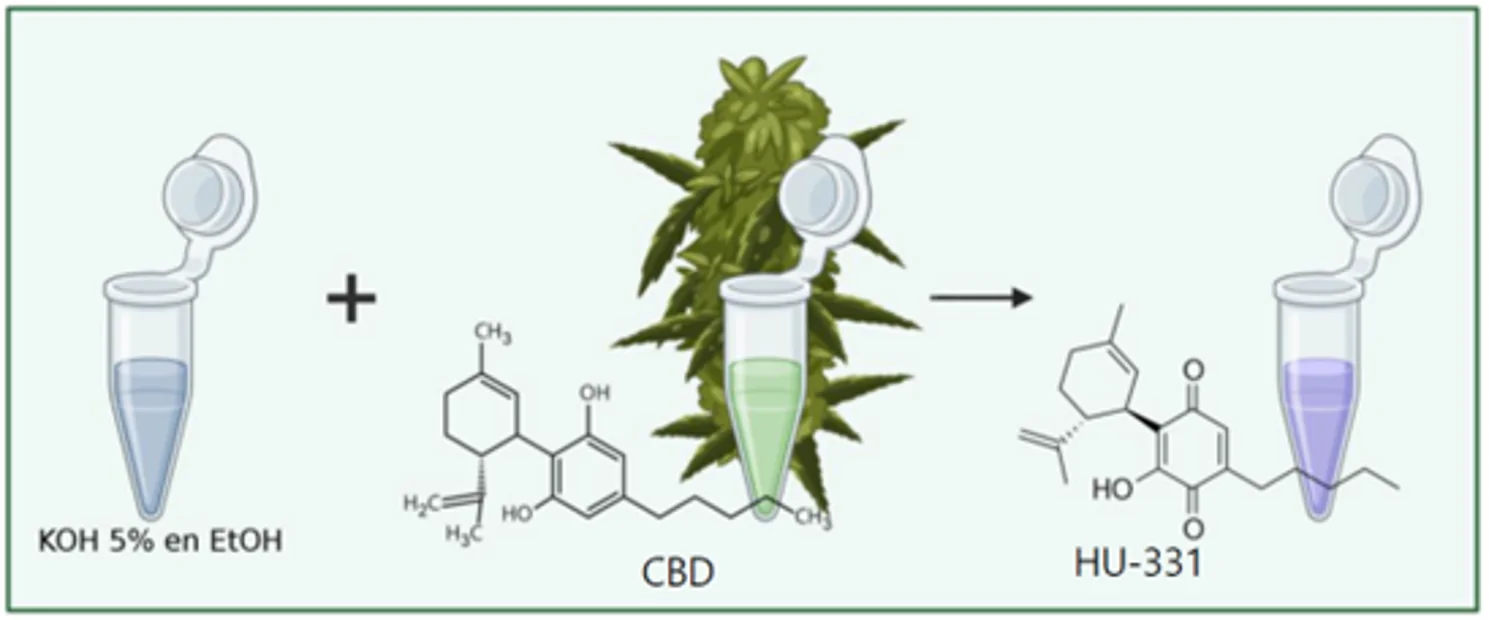
Beam test reaction. CBD in presence of alkaline medium (KOH 5%) forms a violet product, a quinone named HU-331

Historically, colorimetric tests have served as rapid screening tools for cannabis identification (Sofrás 2023, Galand et al. 2004, Liu et al. 2020, Sherma and Rabel 2019, Valdes et al. 2024, Watanabe et al. 1978, Acosta and Almirall 2021, Acosta et al. 2022, Mechoulam et al. 1968). Among these, the Beam reaction, first described by William Beam in 1911, remains one of the most distinctive (Mechoulam et al. 1968, Sudborough and Science 1918). Despite its simplicity, the Beam test exhibits remarkable specificity. Research has demonstrated that among 120 plant species across 28 botanical families, only *Salvia sclarea* and *Rosmarinus officinalis* yield a weakly positive reaction. Furthermore, when tested against 48 natural products, including various monoterpenes and aromatic compounds, only juglone, a naphthoquinone, produced a color change (violet-brown) remotely similar to the intense violet characteristic of the Beam reaction. This high degree of selectivity renders the test a robust candidate for the targeted identification of CBD in complex botanical matrices. The reaction involves the treatment of a cannabis extract with a 5% potassium hydroxide (KOH) solution in ethanol, resulting in a characteristic intense violet coloration (Mechoulam et al. 1968) (Fig. 1). Chemically, it is now understood that this color change is specific to CBD and its precursor, cannabidiolic acid (CBDA), and does not occur with Δ−9-THC. The mechanism involves the oxidation of CBD in an alkaline medium to form a hydroxy-quinone derivative, which acts as a chromophore. This chemical basis of the transformation was further elucidated by Mechoulam’s group (Mechoulam and Gaoni 1967), who determined that the violet chromophore arises from the oxidation of structural portions of the CBD molecule under basic conditions (5% ethanolic KOH) (Mechoulam et al. 1968). This process leads to the formation of two hydroxyquinones. Interestingly, one of these primary oxidation products is HU-331 (3(S)−3’-hydroxy-2’-p-mentha-1,8-dienyl-5’-pentyl-1’,4’-benzoquinone). Beyond its role as a chromophore, HU-331 has gained pharmacological importance for its potent anticarcinogenic activity, acting as a highly effective inhibitor of DNA topoisomerase II even at nanomolar concentrations (Kogan et al. 2007, Regal et al. 2014, Trac et al. 2021).

Despite its long-standing use as a qualitative test in forensic science, its potential for quantitative application has been largely overlooked in the modern era.

This study aims to bridge this gap by validating the Beam reaction as a quantitative tool to mitigate the risks associated with the growing consumption of non-standardized products. By optimizing reaction kinetics and utilizing UV-Vis spectrophotometry, we present a reliable, low-cost, and reproducible method for total CBD quantification. This approach facilitates rapid quality assessment, serving as an accessible quality control solution for dispensaries, pharmacies, and medical clinics by filling the niche between simple qualitative tests and complex chromatographic systems.

## Materials and methods

### Reagents and standards

Purified Cannabidiol (CBD) standard (purity ≥ 98%) was kindly provided by Avicanna. Beam reagent was prepared as a saturated solution of 2% potassium hydroxide (KOH, analytical grade, ≥ 85%, Merck) in ethanol (absolute, ≥ 99.8%). All other solvents used (methanol, hexane, chloroform, methylene chloride) were of analytical grade (purity ≥ 99.5%) and used as received without further purification. Real samples consisted of two commercial cannabis oils (S1 and S2), previously quantified via HPLC for comparative purposes.

### Spectroscopic and kinetic insights into the Beam test quinone product

#### Spectral characterization and wavelength selection

To identify the optimal wavelength for detecting the Beam reaction chromophore, a 1 mg/mL CBD stock solution in methanol was prepared. The reaction was performed at a 7:3 ratio (KOH reagent: CBD solution) to ensure a sufficiently alkaline environment. After a 24-h incubation at room temperature in the dark, the sample was centrifuged to remove precipitates. The supernatant was analyzed in a Jasco V-730 spectrophotometer (SpectraManager software) using a 1 mL quartz cuvette to determine the maximum absorbance (λmax).

#### Reaction kinetics and stability

The reaction kinetics were re-evaluated to establish the optimal measurement timeframe. CBD concentrations (50–300 µg/mL) were monitored at intervals (0, 0.25, 0.5, 1, 2, 4, 6, 8, 10, 12, and 24 h). Long-term stability was assessed with a final measurement at seven days.

#### Structural confirmation via FTIR

To confirm that the violet product corresponds to the hydroxyquinone derivative (HU-331), FTIR analysis was performed. Aliquots of isolated CBD and the Beam reaction product were dried under nitrogen flow. Spectra were recorded in the 4000–500 cm⁻¹ range using an FTIR-Raman Nicolet iS 50 with an attenuated total reflection (ATR) accessory.

#### Determination of the correlation between CBD concentration and final absorbance: linearity range

To assess the linearity of the Beam reaction response as a function of CBD content, a calibration curve was constructed. CBD standard solutions were prepared at concentrations of 50, 100, 150, 200, and 250 µg/mL, maintaining a constant KOH: CBD ratio of 7:3 within a final volume of 2 mL. The reaction mixtures were incubated in the dark after 24 h. Prior to analysis, samples were centrifuged to remove precipitates resulting from KOH saturation. The absorbance of the supernatant was subsequently measured at the wavelength identify in Sect. 2.1.1 (530 nm) using a Jasco V-730 spectrophotometer, controlled by SpectraManager software. Molar absorptivity was calculated according to Lambert-Beer equation and the slope of calibration curve. All assays were performed in triplicate.

### Development of the Beam test- based spectrophotometric method for CBD quantification

#### Matrix effect mitigation and solvent selection

Initial application to cannabis oils (resin diluted in carrier olive oil) revealed significant matrix interference. The lipidic nature of the samples induced micelle formation and phase separation when using methanol. Various organic solvents (chloroform, methylene chloride) were tested, with hexane yielding superior results in terms of miscibility and reaction performance.

#### Recovery assay (matrix spiking)

To validate the extraction efficiency, a matrix spiking assay was designed. 1 mg of CBD was added to 1 mL of blank olive oil and subsequently extracted with hexane. Recovery (R%) was calculated by comparing the quantified amount against the spiked concentration using the standard addition formula.

#### Analysis of real samples (S1 and S2)

Aliquots (100 µL) of commercial oils were diluted in hexane (1:100). Then, 100 µL of this dilution was mixed with 200 µL of hexane and 700 µL of Beam reagent. Reagent handling was performed in an ice bath due to hexane’s volatility, and the reaction proceeded at 4 °C for 24 h before measuring absorbance at 530 nm.

### Validation

The method was developed and validated through linearity, accuracy, precision, detection and quantitation limits, and recovery studies according to the International Conference of Harmonization (ICH) Validation of Analytical Procedures.

#### Preparation of standard solution and linearity

A standard CBD solution in hexane was prepared at a concentration of 1 mg/mL. From this solution, different aliquots were taken to construct a calibration curve in a concentration range of 50 to 250 µg/mL (50, 100, 150, 200, 250 µg/mL) maintaining a constant KOH: CBD ratio of 7:3 within a final volume of 1 mL. The reaction mixtures were incubated in the dark at a temperature of 4 °C (to avoid hexane evaporation). After 24 h, samples were centrifuged to remove precipitates resulting from KOH saturation and then evaluated a 530 nm.

#### Precision

Precision was calculated in terms of the variability in repeatability validation parameters with the following formula:

1 $$\mathrm{RSD}\%=\left(\left(\mathrm{SD}/100\right)/\mathrm X\right)$$

where, SD is the estimate of the standard deviation and x is the average of the replications made.

#### Repeatability

The repeatability (intraday) of the method was evaluated by measuring the same sample (standard solution of 150 µg/mL) three times on the same day, performed by the same operator with the same method and instrument. The repeatability (interday) of the method was evaluated by measuring the sample three times on three different days performed by the same operator with the same method and instrument.

#### Detection and Quantification Limit (LOD and LOQ)

The detection limit (LOD) and quantification limit (LOQ) may be expressed as follows:

2 $$\mathrm{LOD}=\left(3.3\times\mathrm\sigma\right)/\mathrm S$$

3 $$\mathrm{LOQ}=\left(10\times\mathrm\sigma\right)/\mathrm S$$

where, σ = the standard deviation of blank responses and S = the slope of the calibration curve. The slope S may be estimated from the calibration curve of the analyte. The estimate of σ was based on the residual standard deviation of the regression line constructed by the calibration curve containing the cannabinoid analysed in the range of QL.

#### Recovery

Recovery was measured by the method of standard addition. The developed method was used to determine the CBD content before and after spiking different concentration of CBD (50 and 100 µg) to S1. Recovery was expressed as a percentage, R (%), according to the following formula:

4 $$\mathrm R\left(\%\right)=\left(\left(\mathrm{Cf}-\mathrm C\right)/\mathrm{Cc}\right)\times100$$

where, Cf is the endogenous amount of the cannabinoid in the sample plus the amount of standard added to the analyte under examination, C is the endogenous amount present in the sample not added with the standard, and Cc is the amount of the standard analyte added to the sample.

### Sustainability assessment

The environmental impact of the developed method was evaluated using the Analytical Greenness (AGREE) metric software (CITA). The assessment considered the 12 principles of green analytical chemistry, including sample treatment, solvent toxicity, and waste generation, to provide a comprehensive greenness score (Pena-Pereira et al. 2020).

## Results and discussion

### Wavelength selection, linearity, and reaction kinetics

**Fig. 2:**
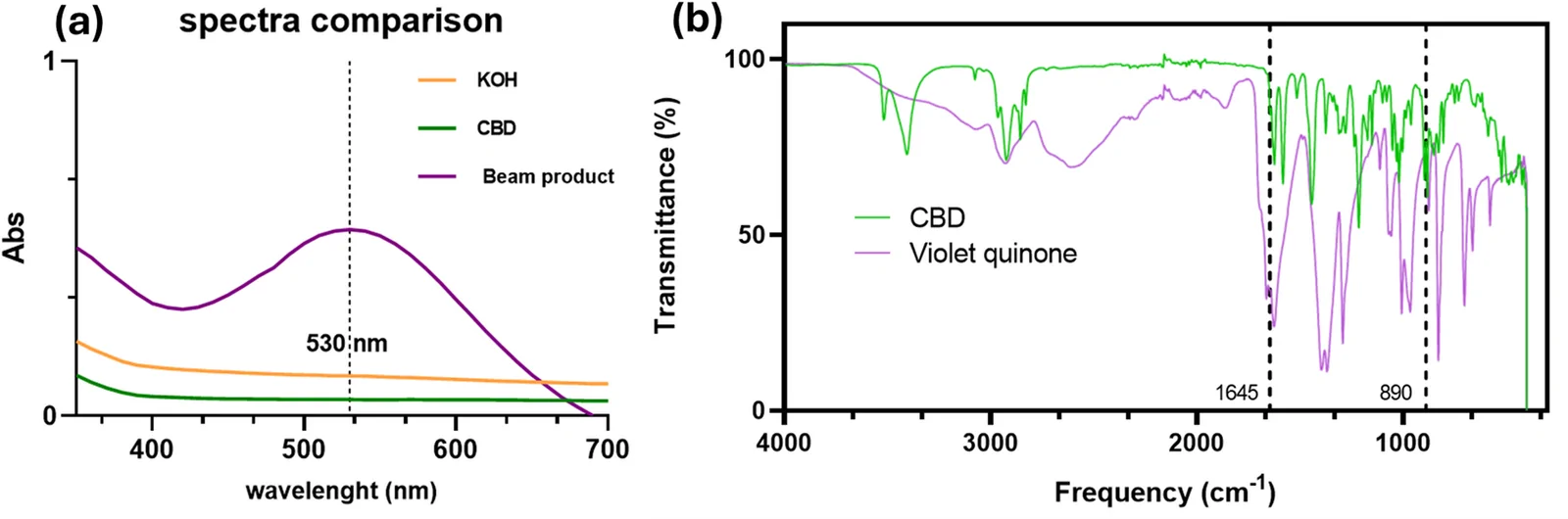
UV spectra of KOH, CBD and Beam product (violet quinone) (**a**). FTIR spectra for CBD and violet product of Beam Test (**b**)

The chemical transformation of cannabidiol (CBD) into a colored product via the Beam reaction was monitored and confirmed using UV-Vis spectrophotometry across the 300–700 nm range. Individual spectra of the precursors revealed that both pure CBD and the Beam reagent (5% KOH in ethanol) exhibit significant absorbance only in the ultraviolet region (specifically below 400 nm), lacking optical activity in the visible spectrum (Fig. 2a). Following the interaction of the components and the incubation period, the spectrum of the reaction product showed the emergence of a broad and well-defined absorption band in the visible region, which was absent in the starting materials. A maximum absorbance wavelength was identified at 530 nm (Fig. 2a), this value correlates with the complementary light absorption required for the perception of the intense violet color characteristic of HU-331-type hydroxyquinones (Mechoulam and Gaoni 1967).

### Structural characterization via FTIR spectroscopy

To elucidate the chemical transformation occurring during the Beam reaction, Fourier-Transform Infrared (FTIR) spectra of pure CBD and the resulting violet chromophore were compared. As shown in Fig. 2b, the infrared profile of the reaction product exhibits significant structural modifications that confirm the oxidative conversion of CBD into a quinone derivative. The most prominent evidence of this transformation is the emergence of a sharp, intense absorption band at 1645 cm^− 1^ in the violet quinone spectrum, which is absent in the pure CBD spectrum. This signal is characteristic of the C = O stretching vibration in a conjugated quinone system, specifically identifying the formation of the carbonyl groups of HU-331 (Fig. 1). Furthermore, the appearance of a distinct peak at 890 cm^− 1^ corresponds to the specific out-of-plane bending vibrations of the quinone ring system, providing definitive proof of the structural rearrangement. Additionally, the spectrum of the reaction product shows a noticeable broadening and shift in the hydroxyl (-OH) region (3200–3500 cm^− 1^), consistent with the transition from the phenolic structure of CBD to the hydroxyquinone form. These spectroscopic markers align perfectly with the historical descriptions of quinone derivatives by Mechoulam et al. (1968) (Mechoulam and Gaoni 1967) confirming that the colorimetric change measured at 530 nm is directly linked to the formation of the bioactive compound HU-331.

### Linearity and quantitative range

**Fig. 3:**
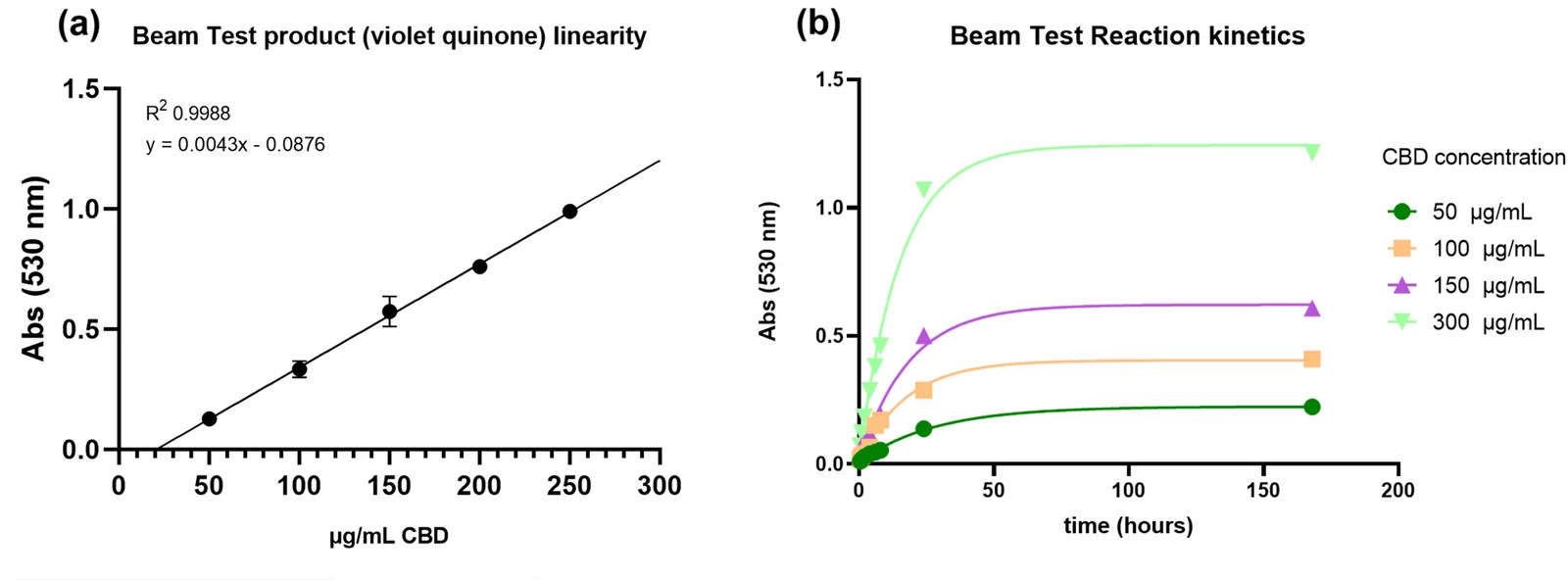
Beam reaction for CBD quantification: linearity of calibration curve (**a**) and Beam Test kinetics (**b**)

The quantitative potential of the optimized Beam reaction was evaluated by constructing a calibration curve using CBD standards in hexane. As shown in Fig. 3a, the method exhibits excellent linearity within the concentration range of 50 to 250 µg/mL. The regression equation was determined to be: y = 0.0043x − 0.0876. The correlation coefficient (R^2^ = 0.9988) demonstrates a robust proportional relationship between CBD concentration and the absorbance measured at 530 nm. This high degree of linearity confirms that the formation of the violet quinone follows the Beer-Lambert law under the established experimental conditions, allowing for reliable quantification within the studied range. From the slope of the calibration curve and using the Lambert-Beer law, the apparent molar absorptivity (ε) of the reaction product was calculated to be 1350 (M cm)^−1^ at 530 nm. This calculation assumed a path length of 1 cm and was based on the molecular weight of CBD (314.47 g/mol).

### Analysis of reaction kinetics and stability

The formation of the violet chromophore was detectable by UV-Vis starting from 30 min for all tested concentrations. Kinetic monitoring established that the most significant increase in absorbance occurred within a 24-hour period (Fig. 3b). Measurements performed one week later showed no significant differences in absorbance values, confirming that the reaction reached a stable plateau. The selection of a 24-h incubation period was selected to ensure that the reaction reaches a full equilibrium state, providing a stable absorbance plateau that minimizes analytical variability and guarantees reproducibility. Additionally, this protocol simplifies batch processing, by eliminating strict time dependence during the early stages of chromophore formation. This stability over time allowed for the establishment of 24 h as the optimal incubation period for all subsequent quantitative analyses. Furthermore, the long-term persistence of the signal for up to seven days provides a significant practical advantage for batch processing in laboratory settings, as the violet product remains stable without degradation for an extended window.

### Method development and optimization

The analytical protocol was systematically optimized to ensure maximum robustness and applicability to real-world samples, evaluating four critical parameters. First, hexane was selected as the extraction and reaction solvent over polar alternatives (e.g., methanol, ethanol). To maintain volume stability and prevent volatilization, the process was conducted at 4 °C. Its superior affinity for lipophilic cannabinoids provided higher extraction efficiency from oil matrices and yielded cleaner analytical signals by minimizing the co-extraction of polar interferents. Second, the alkalinity of the system was refined: while classical Beam tests employ a 5% KOH concentration, we found that reducing it to 2% in the final reaction mixture ensures the robustness of the method by preventing the formation of precipitates that interfere with spectrophotometric accuracy, without compromising the reaction rate. Third, the sample-to-reagent ratio was optimized to provide a molar excess of KOH, ensuring a stable alkaline environment for the complete oxidative conversion of CBD into its stable purple hydroxyquinone form (HU-331). Finally, kinetic studies ("Analysis of reaction kinetics and stability" section) identified that a 24-h incubation provides a stable plateau, ensuring reproducibility across different laboratory settings.

Following these optimizations, the analysis of the spiked matrix samples demonstrated a mean recovery of 98%. These results confirm a highly efficient extraction of CBD from the oil by using hexane, indicating that the method allows for accurate quantification without significant interference from the lipid vehicle.

To validate the proposed method, the CBD content of two commercial oil formulations was quantified and compared against the reference values obtained by HPLC (Table 1). Therefore, two commercial oil formulations were selected based on their legal availability within the current regulatory framework and their distinct composition profiles: S1, a pharmaceutical-grade formulation based on purified CBD resuspended in oil (available in pharmacies), and S2, a widely used imported dietary supplement derived from a whole-hemp extract (full spectrum).

The analysis of the S1 sample revealed a high degree of concordance between the spectrophotometric measurements and the chromatographic reference (Table 1), demonstrating excellent accuracy. This result confirms that for formulations based on purified CBD, the method is highly specific and free from interference. In contrast, for the S2 oil, a slight overestimation and higher dispersion were observed compared to the HPLC data. This difference is likely attributable to the complex matrix of the whole-plant extract, where co-extracted phytochemicals may introduce minor spectral interferences or matrix effects that are absent in the purified isolate.

However, it is noteworthy that even for the complex extract, the reference value remained within the standard deviation range of the spectrophotometric measurements. These findings suggest that while the method exhibits broad utility, it is particularly robust and precise for pharmaceutical preparations based on purified CBD isolates—such as compounded creams, and pharmacy-dispensed oils—which currently represent a significant portion of the regulated market. Thus, despite the intrinsic limitations of a bulk colorimetric reaction compared to the separation efficiency of HPLC, the proposed method offers a valid, accessible, and cost-effective alternative for quality control in these applications.

**Table 1:** CBD quantitation of S1 and S2. Results comparison between UV-vis proposed method and HPLC

	HPLC-CBD quantitation (mg/mL ± SD)	UV-Vis Spectrophotometry -CBD quantitation (mg/mL ± SD)	Standard deviation between methods	Relative accuracy%
S1	65.9 ± 0.01	64.67 ± 6.16	0.87	98
S2	30 ± 0.01	37.37 ± 7.76	5.21	124

### Validated Parameters: linearity, sensitivity, reproducibility and accuracy

Results of this section are resume in Table 2. The linearity of the analytical response was evaluated during the establishment of the Beam’s reaction characteristics. The resulting calibration curve demonstrated excellent linearity over the tested concentration range, with a determination coefficient R^2^ > 0,99. This behaviour confirms that the colorimetric signal generated under the optimized conditions is directly proportional to the CBD concentration, providing a reliable basis for quantification.

In terms of sensitivity, limits of detection (LOD) and quantification (LOQ) were estimated based on the standard deviation of blank responses (Table 2). Results show a remarkable concordance with the limits of the HPLC reference method (Sofrás et al. 2023). It is important to note that this chromatographic reference employs conventional instrumentation and standard analytical columns, differing from the high-efficiency systems often used in high-throughput analysis. This similarity indicates that, despite the simpler instrumentation, the proposed colorimetric assay achieves a detection capability comparable to that of a classical HPLC workflow.

Regarding precision and accuracy, the method demonstrated robust performance. Although intraday coefficients of variation were slightly higher than those observed in the HPLC analysis, they remained well within acceptable limits for manual procedures. Accuracy was further confirmed through recovery assays, where the matrix was spiked with CBD at two different concentration levels. In both cases, the assay yielded a complete recovery (100%) (Table 2), demonstrating that the extraction with hexane is highly efficient and that the analyte is fully recovered from the oily matrix. This confirms that any minor overestimations observed in complex full-spectrum extracts (such as S2) are not due to extraction inefficiency but to minor spectral contributions from the matrix phytochemicals.

**Table 2:** Validation parameters

	LOD (µg/mL)	LOQ (µg/mL)	Intraday (CV%)	Interday (CV%)	Recovery (spiking 50 µg and 100 µg)
UV-Vis method	1.84	5.58	2.88	4.74	100%
HPLC method	1.77	5.33	3.14	2.62	99%

### Sustainability assessment

**Fig. 4:**
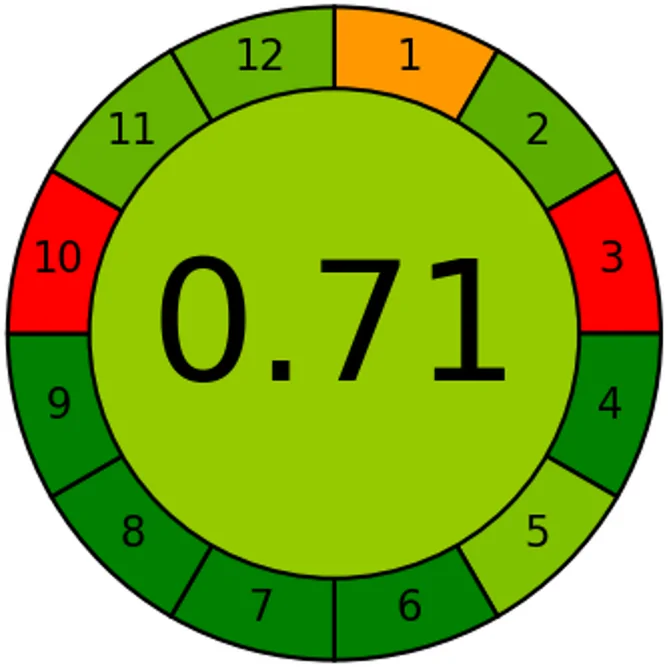
Analytical Greenness (AGREE) pictogram for the optimized Beam spectrophotometric method, showing an overall sustainability score of 0.71

The environmental profile of the method was evaluated using the AGREE metric, yielding a score of 0.71 (Fig. 4). This result reflects a favorable balance between analytical performance and laboratory sustainability. While the use of hexane is required for efficient cannabinoid solubilization, its environmental footprint is minimized through a microscale design (300 µL per sample). This approach offers a practical alternative in cases where high-throughput chromatographic systems may not be required for routine screening. By reducing solvent consumption and simplifying the analytical workflow, the method provides a sustainable and cost-effective option for decentralized laboratories.

### Comparison with other simple analytical methods

The proposed method offers a strategic balance between the simplicity of qualitative screenings and the precision of high-end instrumental techniques. Compared to TLC and HPTLC, which are excellent for multi-component identification, our spectrophotometric approach achieves accurate quantification without requiring expensive densitometric scanning, thus reducing equipment barriers for decentralized laboratories. Furthermore, unlike other common colorimetric assays such as the Fast Blue B test, which often yield transient signals, the optimized Beam protocol provides significantly enhanced chromophore stability.

## Conclusions and future perspectives

This study successfully established an accessible and robust analytical methodology based on the Beam colorimetric reaction for the quantification of Cannabidiol (CBD) in medicinal oils. By transitioning a classic qualitative test into a validated quantitative tool, we have demonstrated that UV-Vis spectrophotometry can offer analytical performance comparable to standard HPLC systems for this specific application. The method exhibited excellent linearity (R^2^ > 0.99) and a limit of quantitation (LOQ) of 5.58 µg/mL, while recovery assays confirmed a near-total extraction efficiency (approx. 100%) from complex lipidic vehicles without significant matrix interference. The application of this technique to commercial samples suggests that while it provides exceptional accuracy for purified CBD formulations, full-spectrum extracts, containing a broader range of secondary metabolites, may introduce minor spectral variations. Consequently, future research should focus on refining sample pre-treatment protocols, such as incorporating solid-phase extraction cleanup steps or mathematical background correction algorithms, to further enhance the method’s accuracy in the presence of diverse botanical matrices. In conclusion, the proposed spectrophotometric method serves as a reliable, cost-effective, and reproducible alternative to traditional chromatography. Indeed, to our knowledge, this work represents the first validated non-chromatographic approach for the reliable quantification of CBD, providing a transformative tool for laboratories without access to high-end instrumentation. By utilizing widely available reagents and instrumentation, this tool is particularly valuable for small-scale pharmacies, dispensaries, and quality control laboratories in resource-limited settings. Implementing such accessible methodologies is a crucial step toward democratizing quality control and mitigating the public health risks associated with unstandardized cannabis-derived products.

## Data Availability

The datasets supporting the conclusions of this article are available in the ScienceDB repository, https://doi.org/10.57760/sciencedb.28115//(https://www.scidb.cn/en/anonymous/amVRUnpp).
